# Time of Day Influences Psychophysical Measures in Women With Burning Mouth Syndrome

**DOI:** 10.3389/fnins.2021.698164

**Published:** 2021-10-01

**Authors:** Janell S. Payano Sosa, Joyce T. Da Silva, Shana A. B. Burrowes, Soo Y. Yoo, Michael L. Keaser, Timothy F. Meiller, David A. Seminowicz

**Affiliations:** ^1^Program in Neuroscience, University of Maryland, Baltimore, Baltimore, MD, United States; ^2^Department of Neural and Pain Sciences, University of Maryland School of Dentistry, Baltimore, MD, United States; ^3^Center to Advance Chronic Pain Research, University of Maryland, Baltimore, Baltimore, MD, United States; ^4^Department of Psychiatry, School of Medicine, Johns Hopkins University, Baltimore, MD, United States; ^5^Section of Infectious Diseases, Department of Medicine, Boston University School of Medicine, Boston, MA, United States; ^6^Montefiore Medical Center, The Bronx, NY, United States; ^7^Oncology and Diagnostic Sciences, University of Maryland School of Dentistry, Baltimore, MD, United States

**Keywords:** burning mouth syndrome, orofacial pain, thermal testing, pressure threshold testing, postmenopause, women, pain, chronic pain

## Abstract

Burning mouth syndrome (BMS) is a chronic orofacial pain condition that mainly affects postmenopausal women. BMS type I patients report little to no spontaneous pain in the morning and increases in pain through the day, peaking in the afternoon. Quantitative sensory testing (QST) findings from BMS type 1 patients are inconsistent as they fail to capture this temporal variation. We examined how QST in BMS type 1 (*n* = 18) compared to healthy participants (*n* = 33) was affected by time of day. QST of the face and forearm included warmth detection threshold (WDT), cold detection threshold (CDT), and heat pain thresholds (HPT), ratings of suprathreshold heat, and pressure pain thresholds (PPT), and was performed twice: once in the morning and once in the afternoon. Compared to healthy participants, BMS patients had higher pain sensitivity to phasic heat stimuli at most temperatures (35°C U = 126.5, *p* = 0.0006, 39°C U = 186.5, *p* = 0.0386, 41°C U = 187.5, *p* = 0.0412, 43°C U = 171, *p* = 0.0167, 45°C U = 168.5, *p* = 0.0146) on the forearm, but no differences in pain thresholds (HPT and PPT) regardless of time of day or body area tested. BMS patients had higher WDT (U = 123, *p* = 0.0172), and lower CDT (U = 98, *p* = 0.0021) of the forearm and lower WDT of the face (U = 55, *p* = 0.0494). The differences in forearm WDT (U = 71.5, *p* = 0.0113) and CDT (U = 70, *p* = 0.0096) were most pronounced in the morning. In summary, BMS type I patients had increased pain sensitivity on the forearm, but no differences in pain thresholds on the face or forearm. Patients also showed altered thermal sensitivity, which depended on body area tested (heightened in the orofacial region but blunted on the forearm), and was more pronounced in the morning plausibly due to hypervigilance.

## Introduction

Burning Mouth Syndrome (BMS) is a chronic orofacial pain condition that mainly affects post-menopausal women (Grushka et al., 1987; Lipton et al., 1993; Albuquerque et al., 2006; Bergdahl and Bergdahl, 2007; Rivinius, 2009; Dahiya et al., 2013). The most prevalent symptom of BMS is burning pain in the oral mucosa including the palate, inside lip, and the tip and anterior two-thirds of the tongue (Lamey, 1996; Abetz and Savage, 2009). However, the affected area of the oral mucosa is clinically normal (Lamey, 1996; Abetz and Savage, 2009). Therefore, in the absence of clear pathology in the oral mucosa, central mechanisms have been suggested to, at least in part, explain the spontaneous burning pain of BMS and the presence of pain in other body regions (Cheung and Trudgill, 2015; Jääskeläinen and Woda, 2017; Lee et al., 2019).

Somatosensory functions in people affected by BMS can be determined psychophysically using quantitative sensory testing (QST) (Madariaga et al., 2020). Previous QST studies have reported mixed results with some reporting BMS patients have higher sensitivity (Grushka et al., 1987; Yang et al., 2019), lower sensitivity (Mo et al., 2015; Hartmann et al., 2017; Kolkka et al., 2019), and no difference in sensitivity (Forssell et al., 2002; Kaplan et al., 2011; de Siqueira et al., 2013; Yilmaz et al., 2016; Watanabe et al., 2019; Honda et al., 2019; Wolowski et al., 2021) of the orofacial region to painful thermal heat stimuli relative to healthy participants (Supplementary Table 1). However, QST studies outside the orofacial region in BMS patients, such as leg and arm extremities, report no differences in sensitivity to painful thermal heat stimuli compared to healthy participants (de Siqueira et al., 2013; Mo et al., 2015; Hartmann et al., 2017; Honda et al., 2019; Watanabe et al., 2019; Wolowski et al., 2021).

These conflicting extra-trigeminal QST findings could be due to the cyclical nature of spontaneous pain in BMS (Cheung and Trudgill, 2015). Therefore, we focus on BMS type I because patients experience little to no pain in the morning and as the day progresses their pain increases peaking in the afternoon (Lamey, 1996; Abetz and Savage, 2009). Thus, if spontaneous pain is related to changes in sensitization, we would expect different QST results at different times of the day in BMS type I. However, this temporal evaluation of somatosensory responses in BMS type I remains unknown.

In the current study, we examined psychophysical responses to thermal and pressure stimuli on the face and forearm in the morning and afternoon in BMS type I patients and healthy participants to address how time of day affects somatosensory responses in BMS type I. We also collected pain diaries from BMS patients across 8 days to illustrate the cyclical nature of pain in this BMS sample. We hypothesized that compared to healthy participants BMS patients have higher pain sensitivity, specific to the orofacial regions, during the afternoon.

## Materials and Methods

### Overview of Data Collection

All research procedures were granted approval by the Institutional Review Board of the University of Maryland, Baltimore. After thorough explanation of the study, informed consent was obtained from all willing participants according to the Declaration of Helsinki.

BMS participants were asked to complete a 2-day experimental session comprised of 3 days of pain diaries followed by test day 1, and test day 2 culminating with 3 more days of pain diaries. We required visit 2 to be within 9 days after visit 1. An option of a 1-day experimental session was offered in order to reduce scheduling conflicts and increase enrollment. The 1-day experimental session comprised of 3 days of pain diaries followed by test day 1 and culminating with 4 more days of pain diaries. Healthy participants from the current study also completed either a 2-day or a 1-day experimental session but they did not complete pain diaries.

### Participants

#### Diagnostic Protocol for Burning Mouth Syndrome Participants

This study took place between the years 2014 and 2018, at the time BMS was a diagnosis of exclusion, i.e., once all possible physical causes of chronic oral mucosal pain/burning sensation are ruled out, patients could be assigned to the Type 1 or Type 2 category. Dental and oral health examinations were performed at the Oral Medicine Program at the University of Maryland School of Dentistry (led by TFM). BMS patients who presented with possible BMS were given a thorough medical history review and oral examination to rule out a specific disease (i.e., herpes) or medical cause (i.e., diabetes, medication) for the pain. A careful review was made looking for hormonal, allergic, salivary gland dysfunction, chronic low-grade trauma and/or psychiatric abnormalities as possible etiologies. Next an oral swab/brush culture was taken to rule out a fungal cause. Blood work request included: CBC with Differential, comprehensive metabolic panel, including fasting glucose, HbA1C, and TSH levels; along with iron, ferritin, folate, vitamin B1, B2, B6, B12, C, magnesium, and zinc levels; plus, testing for HSV 1 and 2 and herpes zoster titers; Lyme’s disease antibody; and H. Pylori antibodies; and finally, Sjogren syndrome panel ANA, anti-SSA/SSB. Additionally, because of our exclusion criteria, potential participants underwent thorough assessment to ensure no other orofacial co-morbidities were present (i.e., TMD, trigeminal neuralgia, etc.) and medical records from their general providers were assessed to ensure no other chronic pain (i.e., chronic back pain, arthritis, etc.) or other types of co-morbidities (i.e., IBS) were present. However, to increase recruitment, we later expanded our inclusion criteria to include BMS participants with co-morbid pain conditions as dictated by a Health History Assessment collected during their participation. We list the results from this assessment in the Results section under Demographics.

Lastly, patients were given a food diary to fill out for 7 days. Patients that were women ages 40–85, peri or postmenopausal, with a presumptive diagnosis of BMS (meaning all oral tissues appear normal on clinical examination), and a consistent circadian pattern of pain, where spontaneous burning pain is absent or minimal in the morning, and moderate to severe in the late afternoon (type 1 patients) were recruited into the study.

#### Recruitment Criteria

BMS patients were recruited following diagnosis at the Oral Medicine Program at the University of Maryland School of Dentistry (led by TFM), where complete dental and oral health examinations were performed. A working diagnosis of BMS was based on a chief complaint of pain or burning in the oral mucosa and/or tongue and exclusion of other known causes of oral burning−like pain. If BMS patients were taking topical medications or in the transition of weaning off a systemic medication, to start a new one, we asked them to come in when they had completely weaned off of the medications and would test them prior to their transition into a new medication regimen. As mentioned above, to increase recruitment, we expanded our inclusion criteria to include BMS participants with co-morbid pain conditions.

Additionally, healthy participants were recruited through campus−wide flyer advertisement and were free of any chronic pain conditions, psychiatric illness, local oral or systemic disease, and salivary dysfunction as dictated by their doctor’s report of medical history.

*Exclusion criteria for all studies:* participants unable or refusing to sign consent for any part of the testing; daily regimen of opiates; excessive alcohol use as measured on the AUDIT (Saunders et al., 1993); or on hormone replacement therapy within the last 30 days. For BMS cohorts if participants were on a systemic medication regimen they were excluded.

#### Enrollment

Informed consent was obtained from each participant according to the Declaration of Helsinki. Altogether, we enrolled 51 total post- or peri- menopausal female participants: 18 BMS patients and 33 healthy participants (Table 1 and Supplementary Table 2).

**TABLE 1:** Number of participants and ages.

All postmenopausal women	Current study		Previous study	Concurrent study	Group totals	
	Healthy group 1	BMS group 1	Healthy group 2	Healthy group 3	Total healthy	Total BMS
Number of participants	11	18	10	12	33	18
Age (SD)	60.8 (± 8.8)	60.6 (± 5.7)	54.9 (± 7.6)	52 (± 3.78)	55.7 (± 7.66)	61.3 (± 6.4)

*SD, standard deviation.*

The healthy control group consisted of pooled data from three separate studies: healthy participant group 1 (*n* = 11) was enrolled in the current protocol with the 18 BMS patients; healthy participant group 2 (*n* = 10) was obtained from deidentified data from a previous study from our laboratory with identical methods for some of the QST procedures; and healthy participant group 3 (*n* = 12) was obtained from deidentified data from a concurrently run study in our laboratory with identical methods for some of the QST procedures.

BMS and control group 1 volunteers presented themselves for a 2-day or a 1-day experimental session. For the 2-day experimental session, we randomized whether a participant would experience a morning (AM) or an afternoon (PM) QST session on the first day and on the second day participants were assigned the opposite time of day (AM/PM) for QST testing. For example, if a participant was given afternoon QST on the first day, they would have QST in the morning of the second day. For participants who could not commit to two testing days we offered a 1-day experimental session, where we randomized whether a participant would experience a morning or an afternoon QST session.

### Diaries

After participants signed the consent form, BMS patients were given an 8-day paper diary to track their oral burning pain intensity and unpleasantness. For the 2-day visit, they completed the diaries for 3 consecutive days prior to the laboratory visit, during the 2-day visit, and for 3 consecutive days after the visit. For the 1-day visit, they completed the diaries for 3 consecutive days prior to the laboratory visit, during the 1-day visit, and for 4 consecutive days after the visit. Participants were asked to rate their burning pain intensity on a scale of 0–10, with 0 meaning “none” and 10 meaning “as bad as you can imagine” and unpleasantness on a scale of 0–10, with 0 meaning “not bothersome” and 10 meaning “extremely bothersome,” at five different time-points: wakeup, 10 a.m., 2 p.m., 6 p.m. and bedtime each day.

### Thermal Testing

Thermal heat and cold stimuli were delivered to the forearm and face via a 27 mm diameter Medoc Pathway CHEPS Peltier thermode with a heating rate of 70°C/s and a cooling rate of 40°C/s (Pain and Sensory Evaluation System, Medoc Advanced Medical Systems Ltd., Ramat Yishai, Israel). Four thermal tests where administered: three tests on the forearm [temperature threshold, “levels,” and “ratings” (not reported here) testing] and one test on the face (temperature threshold testing). The thermode was repositioned along the forearm and cheek after each stimulus to avoid temporal summation.

#### Forearm Temperature Threshold Testing

Participants received a warmth detection threshold (WDT) test, where the temperature increased from a baseline temperature of 32°C at a rate of 1°C/s until mouse click. Then, they received a cool detection threshold (CDT) test, where the temperature decreased from 32°C at a rate of 1°C/s until mouse click. In both WDT and CDT, participants were asked to click the mouse when they first detected a change in temperature. We then tested heat pain threshold (HPT), where temperature increased from 32°C at a rate of 1.5°C/s until mouse click. Participants were asked to press the mouse as soon as the temperature first became painful. WDT, CDT, and HPT were each performed three times and were calculated as the average temperature across the three trials. Subsequently, we created a WDT Total comparison comprised of the AM and PM six WDT exposures (three in the AM and three in the PM) averaged together for each group (BMS vs. healthy). We created a WDT AM comparison the three AM WDT exposures averaged for each group (BMS vs. healthy). The same procedure was followed for the PM comparison. Additionally, we created a WDT AM vs. PM comparison using the three WDT AM averages and the three WDT PM averages for each group (BMS vs. healthy). All of this was repeated for the CDT and HPT tests.

#### Face Temperature Threshold Testing

Following the forearm temperature threshold testing procedures, participants received WDT, CDT, and HPT tests with temperature stimuli presented three times for each test as explained above; however, this time the thermode was placed on the left cheek. The temperature increased (WDT, HPT) or decreased (CDT) from a baseline temperature of 32°C at a rate of 1°C/s until mouse click when they first detected a change in temperature (WDT, CDT) and as soon as the temperature first became painful (HPT). WDT, CDT, and HPT were each performed three times and were calculated as the average temperature across the three trials. Subsequently, we created a WDT Total comparison comprised of the AM and PM six WDT exposures (three in the AM and three in the PM) averaged together for each group (BMS vs. healthy). We created a WDT AM comparison the three AM WDT exposures averaged for each group (BMS vs. healthy). The same procedure was followed for the PM comparison. Additionally, we created a WDT AM vs. PM comparison using the three WDT AM averages and the three WDT PM averages for each group (BMS vs. healthy). All of this was repeated for the CDT and HPT tests.

#### Forearm “Levels” Testing

Subsequently, a “levels” test was administered to the forearm where participants received a series of heat stimuli delivered in ascending order of target temperatures: 35°, 35°, 39°, 41°, 43°, 45°, 47°, and 49°C. We varied the ramp rates to each target temperature in order to maintain the same ramp time of 1.6 s with each heat stimulus, from the baseline temperature of 32°C. Target temperatures were sustained for 6 s, so the total heat stimulus duration including ramps was 9.2 s. A 20 s inter-stimulus interval between target temperatures allowed the participant to input their rating for the presented target temperature. Participants rated pain intensity on a numerical rating scale (NRS) of 0 (no pain) to 10 (extremely intense pain) and pain unpleasantness on an NRS of 0 (not bothersome) to 10 (extremely bothersome pain).

Subsequently, we averaged the first pain intensity and unpleasantness ratings of the first two temperature exposures (35° and 35°C) to obtain a single value for 35°C and leaving us with 7 total temperature exposures in the morning and afternoon (14 temperatures total). Next, we created a pain intensity Total comparison comprised of the AM and PM pain intensity ratings averaged together to obtain a single value per temperature (7 values total) per group (BMS vs. healthy) (see statistical analyses section for more details). The same procedure was followed to create a pain unpleasantness Total comparison per group (BMS vs. healthy). We also created an AM comparison, the 7 temperature stimulations in the AM are averaged together to get a single value per temperature (7 values total) per group (BMS AM vs. healthy AM). The same procedure was followed to create a PM comparison per group (BMS PM vs. healthy PM). We also created an AM vs. PM comparison, were the 7 temperature stimulations in the AM are averaged together to get a single value per temperature (7 values total) and the 7 temperature stimulations in the PM are averaged together to get a single value per temperature (7 values total) per group (BMS AM vs. BMS PM; healthy AM vs. healthy PM). Additionally, the same procedure was followed to create an AM, PM, and AM vs. PM comparison pain unpleasantness rating per group (BMS vs. healthy).

### Pressure Pain Threshold Testing

Bilateral pressure pain thresholds (PPT) were obtained using a Wagner Force Dial tm FDK 10/FDN Series Push Pull Force Gage pressure algometer with a 1 cm^2^ rubber probe tip diameter (20 lbf × 0.25 lbf; 10 kgf × 100 gf). Participants received pressure stimuli at four locations of the body: thumbnails (center of nail plate of the thumbnail avoiding the nailbed), elbows (approximately centered 5 cm away from the lateral epicondyle), temporalis muscle (center of anterior temporalis), and masseter muscle (center of belly of posterior part of masseter found on palpation). Pressure was applied to the left thumbnail, elbow, temporalis, and masseter muscles (repeated three times in that sequential order) and then right thumbnail, elbow, temporalis, and masseter muscles (repeated three times in the listed sequential order). Participants were asked to raise a hand when the pressure first became painful and the pressure at that instant was recorded in kilograms.

Subsequently, we created a PPT Total comparison of the masseter comprised of the AM and PM 12 pressure exposures (left and right masseter) averaged together for each group (BMS vs. healthy). We created a PPT AM comparison of the masseter the six AM pressure exposures (left and right masseter) averaged for each group (BMS vs. healthy). The same procedure was followed for the PM comparison of the masseter. Additionally, we created a PPT AM vs. PM comparison of the masseter using the six PPT AM averages and six PPT PM averages for each group (BMS vs. healthy). The same comparison groups created for the masseter results were followed for the temporalis, elbow, and thumbnail results.

### Statistical Analysis

Because of the small sample size in each group non-parametric tests were performed. In summary, for each type of QST, the following four comparisons were performed due to the variability in sample sizes (Supplementary Table 2 and Table 2): (1) Total comparison: Mann-Whitney *U*-test between groups (healthy vs. BMS) of the average of both time points (AM and PM). For these analyses, all participants in the BMS and healthy groups were included, whether they had data from AM alone, PM alone, or both AM and PM, in which case an average was taken. (2) AM comparison: Mann-Whitney *U*-test between group analyses of data taken from each subject at the AM time point. (3) PM comparison: Mann-Whitney *U*-test between group analyses of data taken from each subject at the PM time point. (4) AM vs. PM comparison: Wilcoxon signed-rank test within group analyses of AM and PM time points within BMS and healthy groups. We also performed an area under the curve (AUC) with respect to ground analysis of pain intensity and unpleasantness ratings for the levels test. AUC was calculated according to the literature following the same grouping as listed above (Pruessner et al., 2003). Because of the small sample size in each group, non-parametric tests were performed. Additionally, separate models were run due to the variation in sample size in each group. Comparisons 2, 3, and 4 were followed by a Bonferroni *post hoc* correction for multiple comparisons with a corrected threshold of *p* = 0.0167.

**TABLE 2:** Comparisons and the statistical tests used.

Comparison type	Comparison name	Description	Statistical test
Between group	Total	[AM + PM of BMS] vs. [AM + PM of healthy]	Mann-Whitney *U*-test
Between group	AM	AM of BMS vs. AM of healthy	Mann-Whitney *U*-test
Between group	PM	PM of BMS vs. PM of healthy	Mann-Whitney *U*-test
Within group	AM vs. PM	AM BMS vs. PM BMS Or AM healthy vs. PM healthy	Wilcoxon signed-rank test

#### Diaries Analyses

Mean, median, and range of BMS pain intensity ratings were reported for each of the 8 days. A Friedman test was used to compare average pain intensity for 5 time points in a day across 8 days, followed by a Dunn’s *post hoc* test for multiple comparisons. No diaries were collected from healthy participants as ratings of burning mouth pain and unpleasantness were presumably zero and therefore no comparisons were made for healthy participants.

#### Face and Forearm Temperature Threshold Analyses

As explained above, participants were presented with three trials within the WDT, CDT, and HPT tests. Therefore, to analyze the WDT Total comparison of the face, all participants in the BMS and healthy groups were included regardless of whether they had data from AM alone, PM alone, or both AM and PM, in which case an average was taken. We averaged the trials within WDT of each group (BMS vs. healthy) to perform Mann-Whitney *U*-test. The same procedure was followed for the CDT Total comparison and HPT Total comparison of the face.

To assess the between group (BMS AM vs. healthy AM) differences in the morning (AM comparison), we performed a Mann-Whitney *U*-test of the data taken from each subject at the AM time point. To assess the between group (BMS PM vs. healthy PM) differences in the afternoon (PM comparison), we performed a Mann-Whitney *U*-test of the data taken from each subject at the PM time point. To assess the within group (BMS AM vs. BMS PM; healthy AM vs. healthy PM) differences in the morning compared to the afternoon (AM vs. PM comparison), we performed a Wilcoxon signed-rank test of the AM and PM time points within BMS and healthy groups. These three group comparisons (AM, PM, and AM vs. PM) were followed by a Bonferroni *post hoc* test for multiple comparisons with a corrected threshold of *p* = 0.0167. The same procedure was followed for the face CDT AM, PM, AM vs. PM comparison and the HPT AM, PM, AM vs. PM comparison.

The same analyses procedure was followed for all WDT, CDT, and HPT forearm comparisons.

#### Forearm “Levels” Analyses

Because of the small sample size in each group non-parametric tests were performed. Additionally, for both intensity and unpleasantness analyses separate models needed to be run for each temperature due to the variation in sample sizes within and between groups; as well as missing data at 45°, 47°, and 49°C between comparison groups in the unpleasantness analyses (see Supplementary Table 2). To analyze the pain intensity Total comparison at 35°C, the AM and PM pain intensity ratings were averaged together per group (BMS vs. healthy) to perform a Mann-Whitney *U*-test. The same procedure for the pain intensity Total comparison was followed for each subsequent temperature 39°, 41°, 43°, 45°, 47°, and 49°C. Additionally, this analysis procedure was followed to create a levels pain unpleasantness Total comparison per group (BMS vs. healthy) for 35°, 39°, 41°, 43°, 45°, 47°, and 49°C.

To assess the between group (BMS AM vs. healthy AM) differences in the morning (AM comparison) at 35°C, we performed a Mann-Whitney *U*-test of the data taken from the average pain intensity rating obtained in the morning. To assess the between group (BMS PM vs. healthy PM) differences in the afternoon (PM comparison) at 35°C, we performed a Mann-Whitney *U*-test of the data taken from the average pain intensity rating obtained in the afternoon. To assess the within group (BMS AM vs. BMS PM; healthy AM vs. healthy PM) differences in the morning compared to the afternoon (AM vs. PM comparison) at 35°C, we performed a Wilcoxon signed-rank test of the averaged AM pain intensity and the averaged PM pain intensity within BMS and healthy groups. These three group comparisons (AM, PM, and AM vs. PM) at 35°C were followed by a Bonferroni *post hoc* test for multiple comparisons with a corrected threshold of *p* = 0.0167. The same procedure for the pain intensity AM, PM, AM vs. PM comparison was followed for each subsequent temperature 39°, 41°, 43°, 45°, 47°, and 49°C followed by a Bonferroni *post hoc* correction for multiple comparisons with a corrected threshold of *p* = 0.0167. Additionally, this analysis procedure was followed for the levels pain unpleasantness AM, PM, AM vs. PM comparison 35°, 39°, 41°, 43°, 45°, 47°, and 49°C followed by a Bonferroni *post hoc* correction for multiple comparisons with a corrected threshold of *p* = 0.0167.

Given repeated exposure to temperatures and correlation between multiple intensity ratings from the levels tests we also performed an area under the curve (AUC) with respect to ground analysis of pain intensity for the levels test. AUC was calculated according to the literature following the same grouping as listed above (Pruessner et al., 2003). Additionally, given the repeated exposure to temperatures and correlation between multiple unpleasantness ratings from the levels tests, the same procedure was followed for AUC with respect to ground analysis of pain unpleasantness for the levels test.

#### Pressure Pain Threshold Analyses

As explained above, participants were presented with pressure a total of 6 times (three times on the left and three times on the right side of the face (temporalis and masseter) and extremities (thumbnail and elbow). To analyze the PPT Total comparison of the masseter, the AM and PM 12 pressure exposures (left and right masseter) were averaged together for each group (BMS vs. healthy) regardless of whether they had data from AM alone, PM alone, or both AM and PM, in which case an average was taken. We then performed a Mann-Whitney *U*-test. The same analyses procedure was followed for the PPT Total comparison of the temporalis, elbow, and thumbnail.

To assess the between group (BMS AM vs. healthy AM) PPT differences in the morning (AM comparison) of the masseter, we performed a Mann-Whitney *U*-test of the average of the six AM pressure exposures (left and right masseter) for each group (BMS vs. healthy). To assess the between group (BMS PM vs. healthy PM) PPT differences in the afternoon (PM comparison) of the masseter, we performed a Mann-Whitney *U*-test of the average of the six PM pressure exposures (left and right masseter) for each group (BMS vs. healthy). To assess the within group (BMS AM vs. BMS PM; healthy AM vs. healthy PM) PPT differences in the morning compared to the afternoon (AM vs. PM comparison) of the masseter, we performed a Wilcoxon signed-rank test using the six PPT AM averages and six PPT PM averages within BMS and healthy groups. These three group comparisons (AM, PM, and AM vs. PM) were followed by a Bonferroni *post hoc* test for multiple comparisons with a corrected threshold of *p* = 0.0167. The same analyses procedures were followed for the PPT AM, PM, and AM vs. PM comparison of the temporalis, elbow, and thumbnail.

## Results

### Demographics

All participants were peri- or post-menopausal women. BMS participants had an age range of 47–74 years (mean 61, SD ± 6). Seventy-seven percent of BMS participants were Caucasian, six percent African American, six percent Asian, and eleven percent mixed race. Healthy participants had an age range of 43–73 years (mean 56, SD ± 8). Seventy-nine percent of healthy participants were Caucasian, seventeen percent African American, and four percent Asian.

Additionally, 10 out of the 18 BMS participants had co-morbid pain conditions. The presence of co-morbidities were as follows: arthritis (*n* = 6), irritable bowel syndrome (*n* = 4), headaches/migraines (*n* = 2), temporomandibular disorder (*n* = 2), foot neuropathy (*n* = 1), fibromyalgia (*n* = 1).

### Diaries

**FIGURE 1:**
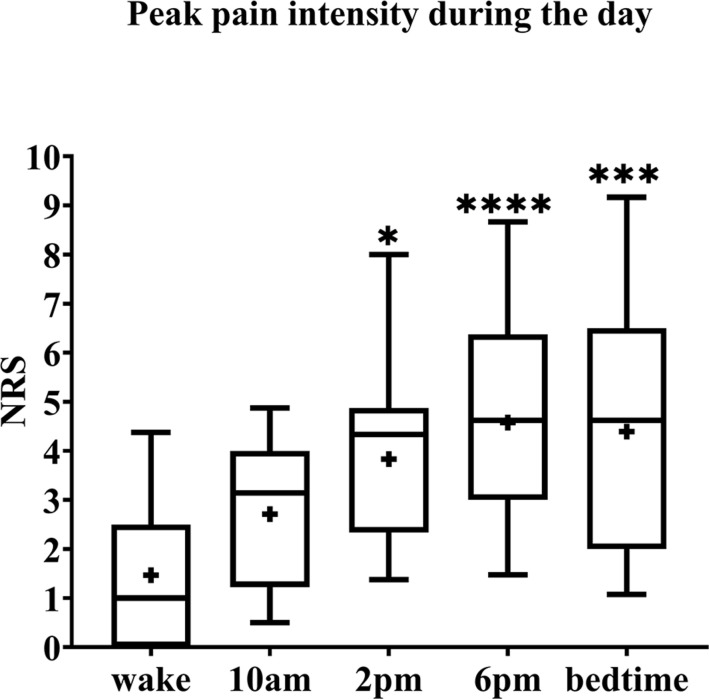
Pain intensity ratings of BMS patients across 8 days. The box spans the interquartile range, whiskers represent the full range, horizontal line within each box mark the median, and the + represents the mean. *p = 0.02, ***p = 0.0003, and ****p < 0.0001 compared to wake. NRS, numerical rating scale.

Pain intensity ratings of BMS patients were significantly higher as the day progressed from wake to bedtime (F_*r*_ = 30.03, *p* < 0.0001) (Figure 1). *Post hoc* analyses revealed pain intensity ratings increased from baseline by 3.3 at 2 p.m. (F_*r*_ = 30.03, *p* = 0.0268), 3.6 at 6 p.m. (F_*r*_ = 30.03, *p* < 0.001) and bedtime (F_*r*_ = 30.03, *p* = 0.0003) compared to wake time. In addition, there were no significant differences in pain intensity ratings across each individual time point and the 8 days of diary recordings; for example, there was no significant difference in pain rating of wake time across the 8 days, no significant difference at 9 a.m. across 8 days, and so on.

### Thermal Testing in Burning Mouth Syndrome vs. Healthy Participants

#### Warmth Detection Thresholds

**FIGURE 2:**
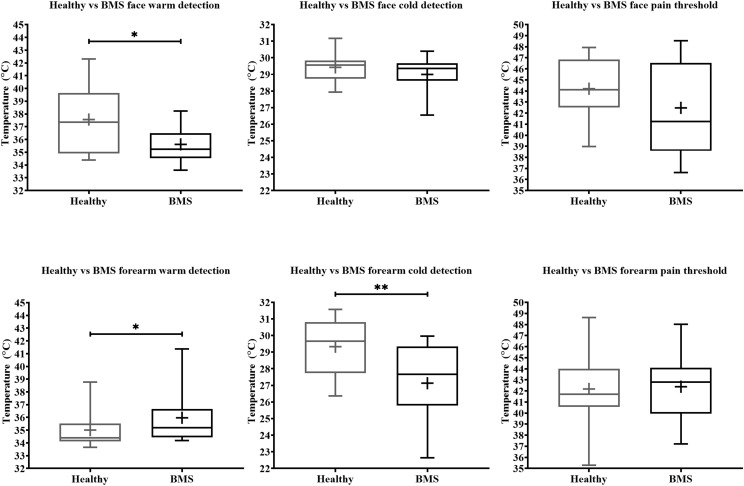
Total comparison of temperature detection and pain threshold in BMS patients compared to healthy participants. WDT, CDT, HPT are shown consecutively in order of exposure to the face (top) and forearm (bottom). *p < 0.05 and **p < 0.005.

**FIGURE 3:**
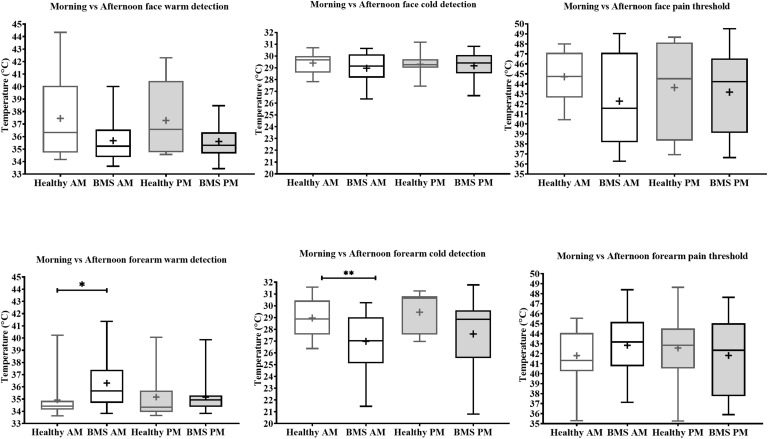
Morning vs. afternoon temperature detection and pain threshold in BMS patients and healthy participants. Face (top) and forearm (bottom) measures. *p < 0.05, **p < 0.005.

Face: In the Total comparison, BMS patients had significantly lower WDTs than healthy participants by 2.2°C (U = 55, *p* = 0.0494) (Figure 2). There were no significant differences in the AM comparison, PM comparison, or AM vs. PM comparison within or between groups (Figure 3).

Forearm: In the Total comparison, BMS patients had significantly higher WDTs compared to healthy participants by 0.8°C (U = 123, *p* = 0.0172) (Figure 2). In the AM comparison, BMS patients had significantly higher WDTs compared to healthy participants by 1.3°C (U = 71.5, *p* = 0.0113). There were no differences in the PM comparison or in the AM vs. PM comparison (Figure 3).

#### Cold Detection Thresholds

Face: BMS patients had no significant differences in CDT compared to healthy participants in the Total comparison (Figure 2). There were no significant differences in AM comparison, PM comparison, or AM vs. PM comparison within or between groups (Figure 3).

Forearm: In the Total comparison, BMS patients had significantly lower CDTs compared to healthy participants by 2°C (U = 98, *p* = 0.0021) (Figure 2). In the AM comparison, BMS patients had significantly lower CDTs compared to healthy participants by 1.9°C (U = 70, *p* = 0.0096). There were no significant differences in the PM comparison, or AM vs. PM comparison within or between groups (Figure 3).

#### Heat Pain Thresholds

Face: BMS patients had no significant difference in HPT compared to healthy participants in the Total comparison (Figure 2). There were no significant differences in AM comparison, PM comparison, or AM vs. PM comparison within or between groups (Figure 3).

Forearm: BMS patients had no significant difference in HPT compared to healthy participants in the Total comparison (Figure 2). In the AM comparison, PM comparison, or AM vs. PM comparison, there were no significant differences within or between groups (Figure 3).

#### “Levels” Forearm Pain Testing Burning Mouth Syndrome vs. Healthy Participants

**FIGURE 4:**
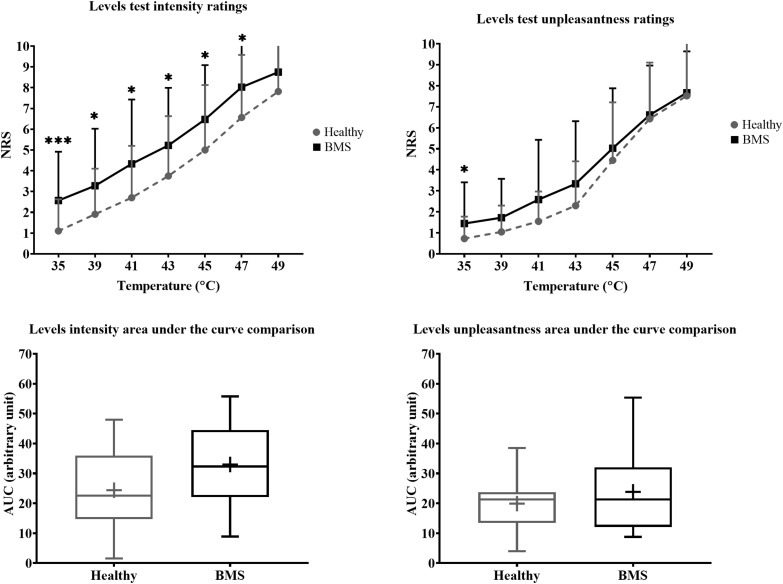
Total comparison of “levels” forearm responses in BMS patients relative to healthy participants. Averaged intensity (left) and unpleasantness (right) responses per temperature and the respective standard deviation. The boxplot (bottom) shows the overall effect of healthy and BMS patients on pain intensity and unpleasantness as AUC. *p < 0.05, ***p < 0.001.

In the Total comparison, BMS participants had significantly higher pain intensity at 35°C by 2.583 pain ratings (U = 126.5, *p* = 0.0006), 39°C by 1.583 pain ratings (U = 186.5, *p* = 0.0386), 41°C by 1.417 pain ratings (U = 187.5, *p* = 0.0412), 43°C by 3 pain ratings (U = 171, *p* = 0.0167), 45°C by 2 pain ratings (U = 168.5, *p* = 0.0146) (Figure 4). In addition, BMS had significantly higher pain unpleasantness at 35°C by 0.583 pain ratings (U = 168.5, *p* = 0.0112) in the Total comparison. There were no differences in the AUC for pain intensity or unpleasantness Total comparison.

**FIGURE 5:**
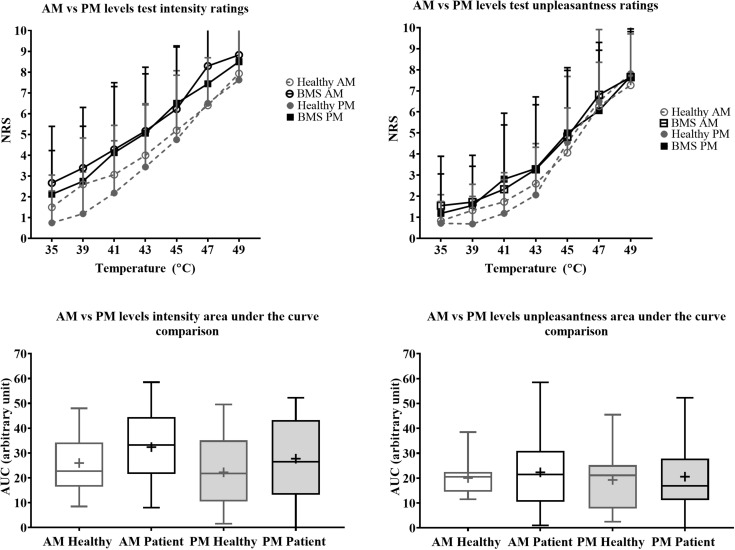
Morning vs. afternoon “levels” forearm responses in BMS patients and healthy participants. Averaged intensity (left) and unpleasantness (right) responses per temperature in the morning (AM) and afternoon (PM) and the standard deviation. The boxplot (bottom) shows the overall effect of healthy and BMS patients on pain intensity and unpleasantness as AUC.

There were no significant differences in pain intensity ratings in the AM comparison, PM comparison, nor in the AM vs. PM comparison. There were also no differences in AUC for pain intensity or pain unpleasantness in the AM comparison, PM comparison, or AM vs. PM comparison (Figure 5).

#### Pressure Testing in Burning Mouth Syndrome vs. Healthy Participants

**FIGURE 6:**
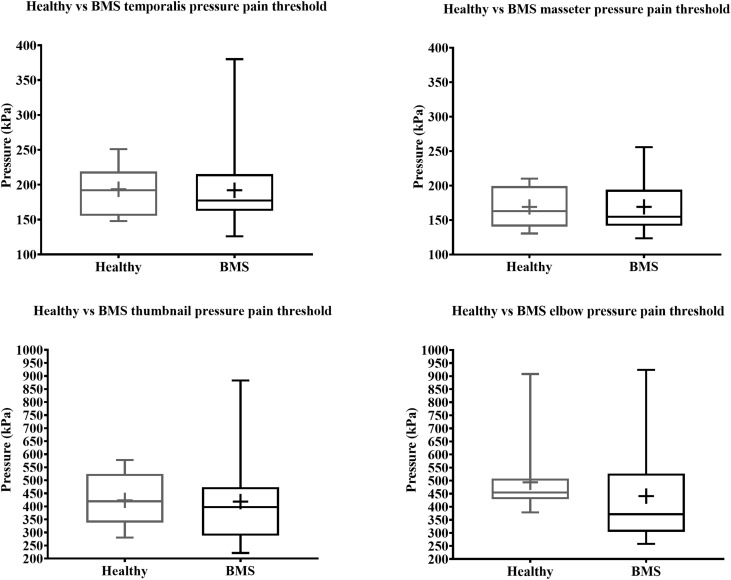
Total comparison of pressure pain thresholds for face (top) and extremity (bottom) of BMS patients and healthy participants.

**FIGURE 7:**
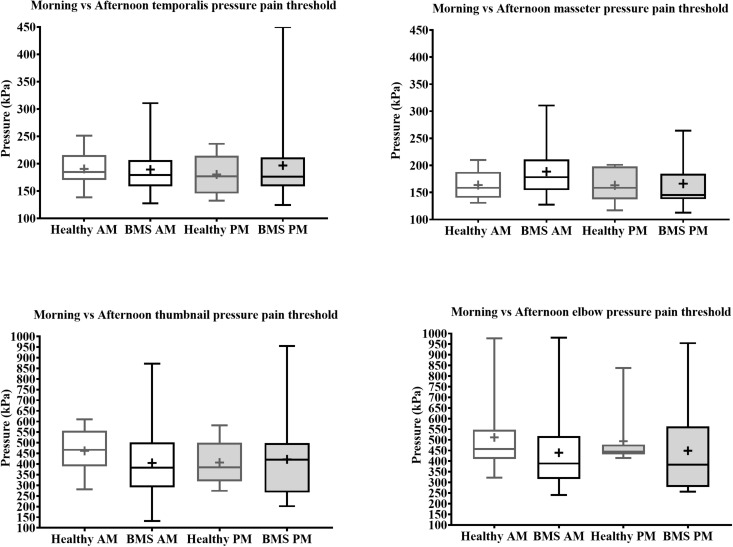
Morning vs. afternoon pressure pain thresholds comparisons for face (top) and extremity (bottom) of BMS patients and healthy participants.

In the Total comparison, BMS patients had no significant differences in PPTs of the masseter, temporalis, thumbnail, and elbow compared to healthy participants (Figure 6). There were no significant differences in PPTs of the masseter, temporalis, thumbnail, and elbow in the AM comparison, PM comparison, or AM vs. PM comparison within or between groups (Figure 7).

## Discussion

In this study, we investigated whether sensory sensitivity of the orofacial region and the forearm was affected by time of day and thus the presence of ongoing pain in BMS type I patients compared to healthy participants. Our hypothesis that compared to healthy participants, BMS type I patients have higher pain sensitivity, specific to the orofacial regions and in the afternoon, was not supported. Our main findings showed that time of day has a significant effect on the spontaneous orofacial pain as quantified by pain diaries. Overall, compared to healthy participants, BMS patients had higher pain sensitivity to phasic heat stimuli (“levels” test) at most temperatures, higher WDT, and lower CDT of the forearm and lower WDT of the face. However, there were no time-dependent differences in experimental thermal pain or pressure pain to the orofacial region. Instead, we found a significant time of day effect for the experimental thermal exposure to the forearm, with BMS patients displaying less sensitivity than controls to both cold and warm temperature detection at only the morning session. This is the first study to compare morning to afternoon QST pain measures in BMS patients compared to healthy participants.

BMS type I is characterized as a burning sensation that is not present upon waking, but which develops in the late morning and progresses during the waking hours, with the greatest intensity of discomfort in the evening (Abetz and Savage, 2009). Based on diary records, we found that spontaneous pain intensity became significantly higher as the day progressed, and the ratings were mostly consistent for each participant across the 8 days of testing. This confirmed the pattern of ongoing pain in BMS type I, i.e., higher pain ratings in the afternoon compared to morning and that pain is present every day (Lamey, 1996; Abetz and Savage, 2009). We also expected that as the day progresses BMS patients would have increased within group orofacial pain and pain sensitivity to other stimuli such as thermal and pressure. However, this within group expectation was not supported as there were no effects of time of day within the BMS group for any other QST measure on any region tested.

We did not find time of day differences between BMS patients and healthy participants in orofacial thresholds assessed by morning and afternoon comparisons of WDT, CDT, HPT, nor masseter muscle or temporalis muscle PPT. Even though the lack of differences could simply be due to our low sample size, Mo et al. (2015) found no differences in WDTs, CDTs, HPTs, and mechanical pain threshold of the tongue, chin, or lip between groups with a comparable sample size of 25 BMS and 19 healthy participants. Thus, enhanced orofacial pain sensitivity in BMS patients may be independent from time of day.

We also found no time-of-day differences in the extremities assessed by thumbnail and elbow PPT comparisons between BMS and healthy participants. Similar to our findings Watanabe et al. (2019) did not find differences in mechanical detection thresholds of the forearm in 28 BMS patients compared to 29 healthy participants, although they did report increased forearm mechanical pain sensitivity. Therefore, a possible interpretation is that BMS does not affect pain evoked by pressure but instead affects mechanical sensitivity of the extremities.

Central sensitization has been suggested as a potential mechanism for the presence of pain in other body regions of BMS patients (Cheung and Trudgill, 2015; Jääskeläinen and Woda, 2017; Lee et al., 2019). We found that BMS patients have lower sensitivity to non-noxious thermal stimulation displayed by the higher WDTs and lower CDTs in the morning at the forearm relative to healthy participants, which does not support a role of central nervous system changes leading to widespread hypersentivity. Instead, the hyposensitivity to cold and warm temperature on the forearm may be due to hypervigilance to their ongoing spontaneous BMS pain as opposed to experimentally evoked thermal stimulation, a phenomenon previously observed in other chronic pain conditions (McDermid et al., 1996; Hollins et al., 2009). Hypervigilance is an enhanced state of sensory sensitivity accompanied by an exaggerated search for threatening information, which may in turn exacerbate the pain experience (Richards et al., 2014; Wermes et al., 2018). Thus, as their BMS pain spontaneously starts to surface in the morning, patients may develop a pain-specific “hypervigilance” to their orofacial pain as a result of continual effort to detect BMS related painful sensations of the orofacial region even in the presence of non-painful cold and warm stimulation on the body. In essence, it can be interpreted that their hypervigilance to the onset of BMS related pain distracts them from the experimentally evoked thermal perception which reflects in lower sensitivity to external innocuous stimuli in the morning.

We found some unexpected outcomes in BMS patients. HPTs tested on the face and forearm in BMS patients did not differ from those in healthy participants. Prior literature on HPTs shows conflicting results, including higher, lower, and non-differing HPTs compared to healthy participants in the orofacial region this while showing no differences in the extremities (Grushka et al., 1987; Forssell et al., 2002; Kaplan et al., 2011; de Siqueira et al., 2013; Mo et al., 2015; Yilmaz et al., 2016; Hartmann et al., 2017; Honda et al., 2019; Kolkka et al., 2019; Watanabe et al., 2019; Yang et al., 2019; Wolowski et al., 2021). It was also unexpected that there were no overall differences in CDTs on the face between groups, regardless of time of the day. Additionally, we did not expect the significantly lower WDTs of the face to no longer be significant in the time of day comparison. Prior literature on CDTs and WDTs are also inconsistent including no differences, higher, and lower CDTs and WDTs on the orofacial region in BMS compared to healthy participants (Supplementary Table 1; Grushka et al., 1987; Forssell et al., 2002; Kaplan et al., 2011; de Siqueira et al., 2013; Mo et al., 2015; Yilmaz et al., 2016; Hartmann et al., 2017; Honda et al., 2019; Kolkka et al., 2019; Watanabe et al., 2019; Yang et al., 2019; Wolowski et al., 2021). Further research is necessary to fully address the contradictory findings in the BMS field and investigate potential mechanisms underlying individual differences between BMS type I patients. We interpret that some of the inconsistent findings in the field could be due to the lack of consideration of the cyclicity of the BMS type I and suggest that incorporating morning and afternoon comparisons can help reduce the variability.

The findings in the present study should be interpreted in light of some limitations. First, sample size was relatively small. Despite our efforts, due to difficulties recruiting healthy participants, we relied on using healthy controls from three separate studies which despite similarities in testing methods can be source of potential biases. Additionally, while we had initially planned to exclude BMS patients with other pain conditions, we modified our inclusion criteria to patients with co-morbid conditions to be able to reach our enrollment target. Given that 55% of our BMS patient sample had comorbid pain conditions, further examination of the effects of overlapping pains is warranted. Note that the presence of one or more other chronic pain conditions occurs in most patients with chronic pain, so our sample of BMS is not out of line with this data (Slade et al., 2020). Second, we were limited by the types of tests we could perform in BMS patients. No intra-oral sensory testing was performed, and we only performed the levels test on the arm in order to prevent triggering BMS discomfort to patients by applying suprathreshold stimuli to the face. Third, we did not have a direct measure to infer central sensitization in BMS patients. Fourth, there is a possibility that differences could exist between BMS patients with perimenopause and menopause, but our sample size does not allow that comparison.

## Conclusion

In conclusion, warm and cold processing is impaired in BMS type I patients, which could suggest hypervigilance toward clinically relevant pain of the orofacial area that results in reduced sensitivity to innocuous stimuli applied to distal body areas. Despite clear increase in spontaneous pain, we saw limited time-of-day dependent effects on QST measures. Subsequent studies should consider potential mechanisms underlying individual differences in BMS type I patients and investigate the impact of pain and other sensory sensitivities in brain signaling in order to further understand BMS symptomatology.

## Data Availability Statement

The raw data supporting the conclusions of this article will be made available by the authors, without undue reservation.

## Ethics Statement

The studies involving human participants were reviewed and approved by the Institutional Review Board of the University of Maryland, Baltimore. The patients/participants provided their written informed consent to participate in this study.

## Author Contributions

TM and DS conceived of and designed the experiment. SY prepared the study protocols and data collection methods. MK provided ongoing technical support. SB advised on non-parametric statistical analyses. SB, JD, DS, and TM all contributed to manuscript preparation. JP performed all data collection, data preprocessing, analysis, and writing of the manuscript. All authors contributed to the article and approved the submitted version.

## Conflict of Interest

The authors declare that the research was conducted in the absence of any commercial or financial relationships that could be construed as a potential conflict of interest.

## Publisher’s Note

All claims expressed in this article are solely those of the authors and do not necessarily represent those of their affiliated organizations, or those of the publisher, the editors and the reviewers. Any product that may be evaluated in this article, or claim that may be made by its manufacturer, is not guaranteed or endorsed by the publisher.
