# Supplementary material for: Time of Day Influences Psychophysical Measures in Women With Burning Mouth Syndrome
Source: Front Neurosci. 2021 Oct 1;15:698164. doi: 10.3389/fnins.2021.698164 (PMC8519262; doi:10.3389/fnins.2021.698164)
Supplement: Supplementary file 1 [file Table_1.docx]

**Supplementary Tables**

Supplementary Table 1: Literature review findings summary. ^↑^BMS higher than healthy. ^↓^BMS lower than healthy. ^–^no difference between groups. WDT: warm detection threshold, CDT: cold detection threshold, HPT: heat pain threshold, PPT: pressure pain threshold. *note general list of results listed, for more details please refer to the publications directly.

| Publication | Orofacial or extremities | Temp or pressure | WDT | CDT | HPT | mechanical pain sensitivity | Mechanical detection threshold |
| --- | --- | --- | --- | --- | --- | --- | --- |
| Grushka et al., 1987 | orofacial | both | - | - | - HPT  $\downarrow$ heat pain tolerance | N/A | N/A  - 2-point discrimination |
| Forsell et al., 2002 | orofacial | temp | $\uparrow$ | $\uparrow$ | - | N/A | N/A |
| Kaplan et al., 2011 | orofacial | temp | - | - | - | N/A | N/A |
| de Siqueira et al., 2013 | both | both | - | $\uparrow$ orofacial  -extremities | - | - | - |
| Mo et al., 2015 | both | both | $\uparrow$ orofacial  -extremities | $\downarrow$ orofacial  -extremities | $\uparrow$ orofacial  -extremities | - | - |
| Yilmaz et al., 2016 | orofacial | both | $\downarrow$ | $\uparrow$ | - | - | - |
| Hartmann et al., 2017 | both | both | $\uparrow$ orofacial  -extremities | $\uparrow$ orofacial  -extremities | $\uparrow orofacial$  -extremities | - | - |
| Watanabe et al., 2019 | both | both | - | - | - | - orofacial  $\uparrow$ extremities  (<6months  with BMS) | $\uparrow$ orofacial  (<6months  with BMS)  - extremities |
| Yang et al., 2019 | orofacial | both | $\uparrow$ | $\uparrow$ | $\downarrow$ | $\downarrow$ | $\uparrow$ |
| Kolkka et al., 2019 | orofacial | temp | $\uparrow$ | $\uparrow$ | $\uparrow$ | N/A | N/A |
| Honda et al., 2019 | both | both | - | - | - | N/A  -Filament prick pain detection threshold | N/A  -Tactile detection threshold |
| Wolowski et al, 2021 | both | temp | - | - | - HPT  $\uparrow$ orofacial cold pain tolerance | N/A | N/A |
| Our totals | both | both | $\downarrow$ orofacial  $\uparrow$extremeties | - orofacial  $\downarrow extremities$ | - orofacial  - extremities | - orofacial PPT  - extremities PPT | N/A |
| Our AM vs PM | both | both | - orofacial  $\uparrow$extremeties AM | -orofacial  $\downarrow extremities$  AM | - orofacial  - extremities | - orofacial PPT  - extremities PPT | N/A |

Supplementary Table 2: number of participants in each test and comparison. *represents exceptions where number of participants was lower by one participant than stated in table due to missing data: at * AM levels intensity had an n=17 BMS at 47°C and at 49°C; at * AM Levels unpleasantness had an n=14 healthy and an n=16 BMS at 47°C; and an n=17 BMS at 45°C and 49°C.

| **Comparison** | **Arm thermal detection** | | **Face thermal detection** | | **Pressure** | | **“Levels”**  **intensity** | | **“Levels” unpleasantness** | | **Diaries** |
| --- | --- | --- | --- | --- | --- | --- | --- | --- | --- | --- | --- |
|  | **healthy** | **BMS** | **healthy** | **BMS** | **healthy** | **BMS** | **healthy** | **BMS** | **healthy** | **BMS** | **BMS** |
| **Total** | **23** | **18** | **11** | **18** | **11** | **18** | **33** | **18** | **33** | **18** | **15** |
| **AM** | **16** | **18** | **9** | **18** | **9** | **18** | **15** | **18*** | **15*** | **18*** | **0** |
| **PM** | **17** | **16** | **8** | **16** | **8** | **16** | **16** | **16** | **16** | **16** | **0** |
| **AM vs PM** | **9** | **16** | **6** | **16** | **6** | **16** | **9** | **16** | **9** | **16** | **0** |
